# Recombinant chimpanzee adenovirus AdC7 expressing dimeric tandem-repeat spike protein RBD protects mice against COVID-19

**DOI:** 10.1080/22221751.2021.1959270

**Published:** 2021-08-12

**Authors:** Kun Xu, Yaling An, Qunlong Li, Weijin Huang, Yuxuan Han, Tianyi Zheng, Fang Fang, Hui Liu, Chuanyu Liu, Ping Gao, Senyu Xu, Xueyuan Liu, Rong Zhang, Xin Zhao, William J. Liu, Yuhai Bi, Youchun Wang, Dongming Zhou, Qinghan Wang, Wenli Hou, Qianfeng Xia, George F. Gao, Lianpan Dai

**Affiliations:** a Key Laboratory of Tropical Translational Medicine of Ministry of Education, School of Tropical Medicine and Laboratory Medicine, The First Afﬁliated Hospital, Hainan Medical University, Haikou, People’s Republic of China; b Research Network of Immunity and Health (RNIH), Beijing Institutes of Life Science, Chinese Academy of Sciences, Beijing, People’s Republic of China; c Savaid Medical School, University of Chinese Academy of Sciences, Beijing, People’s Republic of China; d Chengdu Kanghua Biological Products Co., Ltd, Chengdu, People’s Republic of China; e Division of HIV/AIDS and Sex-Transmitted Virus Vaccines, Institute for Biological Product Control, National Institutes for Food and Drug Control (NIFDC), Beijing, People’s Republic of China; f Laboratory of Animal Infectious Diseases, College of Animal Sciences and Veterinary Medicine, Guangxi University, Nanning, People’s Republic of China; g School of Public Health, Cheeloo College of Medicine, Shandong University, Jinan, People’s Republic of China; h CAS Key Laboratory of Pathogen Microbiology and Immunology, Institute of Microbiology, Chinese Academy of Sciences, Beijing, People’s Republic of China; i CAS Center for Inﬂuenza Research and Early-Warning (CASCIRE), CAS-TWAS Center of Excellence for Emerging Infectious Diseases (CEEID), Chinese Academy of Sciences, Beijing, People’s Republic of China; j National Institute for Viral Disease Control and Prevention, Chinese Center for Disease Control and Prevention, Beijing, People’s Republic of China; k Department of Pathogen Biology, School of Basic Medical Sciences, Tianjin Medical University, Tianjin, People’s Republic of China

**Keywords:** COVID-19, SARS-CoV-2, vaccine, adenovirus, antibody, immune response

## Abstract

A safe and effective vaccine is urgently needed to control the unprecedented COVID-19 pandemic. Four adenovirus-vectored vaccines expressing spike (S) protein have been approved for use. Here, we generated several recombinant chimpanzee adenovirus (AdC7) vaccines expressing S, receptor-binding domain (RBD), or tandem-repeat dimeric RBD (RBD-tr2). We found vaccination via either intramuscular or intranasal route was highly immunogenic in mice to elicit both humoral and cellular immune responses. AdC7-RBD-tr2 showed higher antibody responses compared to either AdC7-S or AdC7-RBD. Intranasal administration of AdC7-RBD-tr2 additionally induced mucosal immunity with neutralizing activity in bronchoalveolar lavage fluid. Either single-dose or two-dose mucosal administration of AdC7-RBD-tr2 protected mice against SARS-CoV-2 challenge, with undetectable subgenomic RNA in lung and relieved lung injury. AdC7-RBD-tr2-elicted sera preserved the neutralizing activity against the circulating variants, especially the Delta variant. These results support AdC7-RBD-tr2 as a promising COVID-19 vaccine candidate.

## Introduction

As of 29 March 2021, the pandemic of COVID-19 has accounted for more than 126 million laboratory-confirmed cases, with approximately 2.8 million deaths worldwide [1,2]. The outbreak is still growing rapidly worldwide. Besides, the emergence of variants raised global concern for the efficacy of current vaccines. Multiple platforms have been used to develop vaccines against COVID-19 [3], including inactivated vaccine [4–8], live attenuated vaccine [9], protein subunit [10–12], virus-like particles [13], virus vectored vaccine [14–19], mRNA [20–22], and DNA [23,24]. Both human and chimpanzee adenovirus vectors were widely used for vaccine development. So far, four adenovirus-vectored vaccines against COVID-19 have been approved for emergency use, including ChAdOx1 nCoV-19 (chimpanzee adenovirus type Y25 vector, University of Oxford and AstraZeneca) [15], Ad5-nCoV (human adenovirus type 5 vector, Beijing Institute of Biotechnology and CanSino Biological Inc.) [19], Gam-COVID-Vac (human adenovirus type 26 and human adenovirus type 5 vectors, Gamaleya Research Institute) [14], and Ad26.COV2.S (human adenovirus type 26 vector, Janssen Pharmaceutical Companies) [17].

Spike (S) protein is comprised of S1 and S2 subunit, in which, the receptor-binding domain (RBD) of S1 is responsible for recognizing and engaging its host cellular receptor protein angiotensin-converting enzyme 2 (ACE2), and S2 accounts for membrane fusion of virus and host cell [25,26]. Therefore, S protein is a major target for the COVID-19 vaccine. ChAdOx1 nCoV-19, Ad5-nCoV, and Gam-COVID-Vac express full-length S protein of SARS-CoV-2 [14,27–29]. Ad26.COV2.S express full-length S protein with four mutations (R682S, R685G, K986P, and V987P) to stabilize the pre-fusion conformation of S protein, which induced higher neutralizing antibodies and confer better protection than recombinant Ad26 expressing wild-type S [17]. These four vaccines were all administered through the intramuscular (i.m.) route in clinical trials [14,15,19,30]. In addition, ChAd-SARS-CoV-2-S is a simian type 36 adenovirus-vectored vaccine expressing full-length S with substitutions K986P and V987P and conferred mice protection and almost entirely prevented SARS-CoV-2 infections in both upper and lower respiratory tracts through the intranasal (i.n.) route [16].

To control the unprecedented COVID-19 pandemic, a system-wide vaccine pipeline with different platforms, targets, vectors, and different action mechanisms are needed. Since weakly- or non-neutralizing antibodies are believed as the cause of antibody-dependent enhancement (ADE) during both flavivirus and coronavirus infections, vaccine design to minimize the induction of ADE-prone antibodies should be taken into account [31,32]. An RBD-based vaccine is aiming to reduce the potential ADE risk [33–35]. Since most of the potent neutralizing antibodies are against RBD of SARS-CoV-2 [36–38], RBD is an attractive vaccine target. Recombinant SARS-CoV-2 RBD protein vaccines were reported to elicited high neutralizing antibodies in both animals and humans [10,11,33,39]. Besides, previous studies showed that sera from animals vaccinated with SARS-CoV.S protein could exacerbate virus infection *in vitro* through ADE [40,41]. Therefore, we sought to develop COVID-19 vaccines based on RBD.

Here, we developed virus vectored vaccines based on chimpanzee adenovirus type 7 (AdC7), a rare serotype in the human population with the advantage of low level of pre-existing immunity [42,43]. We used a tandem-repeat RBD-dimer (RBD-tr2) as an antigen to increase the immunogenicity. This design has been used in our protein subunit COVID-19 vaccine that has been approved for emergency use in China and Uzbekistan [11,39]. Our adenovirus-based vaccines are aiming to further enhance the T cell responses. AdC7 expressing full-length or monomeric RBD was generated for comparison. Vaccination via *i.m.* and *i.n.* routes was evaluated to dissect the systemic and mucosal immune responses, with the latter are believed to be beneficial for protection in the respiratory system [44]. These results will provide crucial guidance for further clinical trials.

## Materials and methods

### Study design

This study was designed to characterize the immunogenicity and protection efficacy of AdC7-RBD-tr2 in BALB/c mice. Three recombinant AdC7 vaccine candidates expressing SARS-CoV-2 full-length S, RBD, and RBD-tr2 were constructed. The induced humoral and cellular responses were evaluated and compared in BALB/c mice for these three AdC7 vaccine candidates. Besides, the mucosal immune responses were evaluated in mice through i.m. or i.n. immunization. To generate a mouse infection model, the BALB/c mice were transduced with recombinant adenovirus 5 expressing human ACE2 (Ad5-hACE2) via the i.n. route. Five days later, the mice were challenged with SARS-CoV-2. The viral load titration and histopathological analysis of mice lung tissues were performed to evaluate the protection effect of the vaccine candidate. Group sizes were selected on the basis of our previous experience with vaccine designation and evaluation. These mice were randomly distributed between groups.

### Cells, viruses, and animals

Human embryonic kidney 293 (HEK293) cells (ATCC CRL-1573), HEK293 T cells (ATCC CRL-3216), Huh7 hepatoma cells (Institute of Basic Medical Sciences, CAMS), VERO cells (ATCC CCL81), and VERO-E6 were all maintained in complete Dulbecco’s modiﬁed Eagle’s medium (DMEM, Invitrogen, USA) supplemented with 10% foetal bovine serum (FBS) and incubated at 37°C under 5% CO_2_. SARS-CoV-2 (hCoV-19/China/CAS-B001/2020, GISAID No. EPI_ISL_514256-7) was propagated in VERO-E6 cells and titrated by tissue culture infectious dose 50 (TCID_50_) assay on VERO-E6 cells. Speciﬁc pathogen-free (SPF) 6–8 weeks old female BALB/c mice were purchased from Beijing Vital River Laboratory Animal Technology Co., Ltd. (licensed by Charles River), and housed under SPF conditions in the laboratory animal facilities at Institute of Microbiology, Chinese Academy of Science (IMCAS). All animals were allowed free access to water and standard chow diet and provided with a 12-hour light and dark cycle. All animal experiments were approved by the Committee on the Ethics of Animal Experiments of the IMCAS, and conducted in compliance with the recommendations in the Guide for the Care and Use of Laboratory Animals of the IMCAS Ethics Committee.

### Construction and production of recombinant chimpanzee adenovirus

An E1- and E3-deleted, replication-deﬁcient recombinant chimpanzee type 7 adenovirus (AdC7) vector [45] was used to construct recombinant AdC7 vaccines encoding full-length S, RBD, or RBD-tr2 of SARS-CoV-2 (GenBank accession number YP_009724390). The full-length S construct contains MERS S protein signal peptide (MIHSVFLLMFLLTPTES) and amino acids 16 to 1273 of the S protein of SARS-CoV-2. The RBD construct contains the same signal peptide and amino acids 319 to 537 of the S protein of SARS-CoV-2. The RBD-tr2 construct contains the same signal peptide and two RBD (amino acids 319 to 537 of the S protein) connected as tandem repeat without any linker sequence. The cassette of full-length S, RBD, and RBD-tr2 was cloned into pAdC7, forming recombinant adenovirus genome, respectively. These recombinant adenovirus genomes were linearized and transfected into HEK293 cells to rescue the recombinant adenovirus, which was further propagated and puriﬁed by caesium chloride density gradient centrifugation as previously described [46]. To carry out the pre-clinical and clinical studies, AdC7-RBD-tr2 was propagated and purified under Good Manufacturing Practices (GMP) by OBiO Technology (Shanghai) Corp., Ltd. The AdC7-RBD-tr2 (GMP-grade) was used for assays shown in Figure 5 and Supplementary Figures 4, 6, and 7.

### Western blot

HEK 293T cells were pre-plated in a 6-well plate, followed by infected with 1 × 10^9^ vp of AdC7-S, AdC7-RBD, AdC7-RBD-tr2, or empty vector AdC7 (AdC7-empty) as a sham control. Forty-eight hours post infection, cells were lysed, and culture supernatants were collected. Protein samples were separated by 12% SDS-PAGE and analysed by Western blotting with rabbit anti-RBD of SARS-CoV-2 polyclonal antibody. Goat anti-rabbit IgG-horseradish peroxidase (HRP) antibodies were used as secondary antibodies. The membranes were developed by the SuperSignal West Pico chemiluminescent substrate (Thermo Fisher Scientiﬁc, USA).

### Expression and purification of proteins

Monomer RBD protein of SARS-CoV-2 was expressed and purified as previously described [11]. Briefly, signal peptide sequence of MERS-CoV S protein (MIHSVFLLMFLLTPTES) was added to the RBD protein (S protein 319-541, GenBank: YP_009724390) N terminus for protein secretion, and a hexa-His tag was added to the C terminus to facilitate further puriﬁcation processes. The coding sequence was codon-optimized for mammalian cell expression and synthesized by GENEWIZ, China. Then, the construct was cloned into the pCAGGS vector and transiently transfected into HEK 293T cells. After 3 days, the supernatant was collected and soluble protein was puriﬁed by Ni afﬁnity chromatography using a HisTrap™ HP 5 mL column (GE Healthcare). The sample was further puriﬁed via gel ﬁltration chromatography with HiLoad^®^ 16/600 Superdex^®^ 200 pg (GE Healthcare) in a buffer composed of 20 mM Tris-HCl (pH 8.0) and 150 mM NaCl.

### Immunization

AdC7-S, AdC7-RBD, and AdC7-RBD-tr2 were diluted in PBS. AdC7-empty was used as a sham control. Female BALB/c mice at 6–8 weeks of age were immunized with vaccine candidate or sham control through the i.m. or i.n. route. The second dose was the same as the first dose and was given 28 days post-prime vaccination. The sera were collected as indicated in ﬁgures legends.

### Enzyme-linked immunosorbent assay (ELISA)

Binding properties of murine sera to monomer RBD or S protein were determined by ELISA. 96-well plates (3590; Corning, USA) were coated overnight with 3 μg/mL of monomer RBD or S protein (Sino Biological, China) in 0.05 M carbonate–bicarbonate buffer, pH 9.6, and blocked in 5% skim milk in PBS. Serum or bronchoalveolar lavage fluid (BALF) samples were serially diluted and added to each well. The plates were incubated for 2 h and then washed. The plates were incubated with goat anti-mouse IgG-HRP antibody (Abcam, ab6789, for IgG titration), goat anti-mouse IgA-HRP antibody (Abcam, ab97235, for IgA titration), goat anti-mouse IgG1-HRP antibody (Abcam, ab97240, for IgG1 titration), or goat anti-mouse IgG2a-HRP antibody (Abcam, ab97245, for IgG2a titration), incubated for 1.5 h and then washed. The plates subsequently developed with 3,3’,5,5’-tetramethylbenzidine (TMB) substrate. Reactions were stopped with 2 M hydrochloric acid, and the absorbance was measured at 450 nm using a microplate reader (PerkinElmer, USA). The endpoint titres were deﬁned as the highest reciprocal dilution of serum to give an absorbance greater than 2.5-fold of the background values. Antibody titre below the limit of detection was determined as half the limit of detection.

### Pseudotyped virus neutralization assay

SARS-CoV-2 pseudotyped virus preparation and neutralization assay were carried out by a previously published method [47], with some modiﬁcations. Brieﬂy, mice sera or BALF samples were 2-fold serially diluted and incubated with an equal volume of 100 TCID_50_ pseudotyped virus at 37°C for 1 h. The medium was mixed with pseudotyped virus as control. Then the mixture was transferred to pre-plated Huh7 cell monolayers in 96-well plates. After incubation for 24 h, the cells were lysed and luciferase activity was measured by the Luciferase Assay System (Promega, USA) according to the manufacturer’s protocol. Neutralization titre (NT_90_) was deﬁned as the highest reciprocal serum dilution at which the relative light units (RLUs) were reduced by greater than 90% compared with virus control wells. NT_90_ below the limit of detection was determined as half the limit of detection.

### Live SARS-CoV-2 neutralization assay

The neutralizing activity of mouse sera was assessed using a previously described SARS-CoV-2 neutralization assay [11]. Brieﬂy, sera from immunized mice were 4-fold serially diluted and mixed with the same volume of SARS-CoV-2 (100 TCID_50_, hCoV-19/China/CAS-B001/2020, GISAID No. EPI_ISL_514256-7), incubated at 37°C for 1 h. Thereafter, a 100 μL virus-serum mixture was transferred to pre-plated VERO-E6 cells in 96-well plates. Inoculated plates were incubated at 37°C for an additional 72 h, following which the cytopathic effect was observed microscopically. The neutralization titre was deﬁned as the reciprocal of serum dilution required for 50% neutralization of viral infection. All the live virus neutralization assays were conducted under biosafety level 3 (BSL3) facility in IMCAS.

### Titration of neutralizing activity against variants

The pseudotyped virus displaying SARS-CoV-2 (Wuhan-1 reference strain and variant strains) S protein express GFP in infected cells. They were prepared as previously described [48]. Mice sera were 2-fold serially diluted and incubated with pseudotyped virus at 37°C for 1 h. Then the mixture was transferred to pre-plated VERO cell monolayers in 96-well plates. After incubation for 15 h, the transducing unit numbers were calculated on a CQ1 confocal image cytometer (Yokogawa). Neutralization titre was determined by ﬁtting nonlinear regression curves using GraphPad Prism and calculating the reciprocal of the serum dilution required for 50% neutralization of infection. Neutralization titre below the limit of detection was determined as half the limit of detection. In this study, the GFP-encoding pesudotyped virus was only used in the comparison of neutralizing activity against Wuhan-1 reference strain and variant strains.

### AdC7 and human Ad5 neutralization assay

Recombinant Ad5 expressing GFP (Ad5-GFP) and recombinant AdC7 expressing GFP (AdC7-GFP) were used in neutralizing activity titration. Sera from immunized mice were 2-fold serially diluted and mixed with the same volume of Ad5-GFP or AdC7-GFP (200 TCID_50_), incubated at 37°C for 1 h. Thereafter, a 100 μL virus-serum mixture was transferred to pre-plated HEK 293 cells in 96-well plates. Inoculated plates were incubated at 37°C for an additional 15 h, following which the transducing unit numbers were calculated on a CQ1 confocal image cytometer (Yokogawa). Neutralization titre was determined by ﬁtting nonlinear regression curves using GraphPad Prism and calculating the reciprocal of the serum dilution required for 50% neutralization of infection. Neutralization titre below the limit of detection was determined as half the limit of detection.

### Enzyme-linked immunospot (ELISpot) assay

To detect antigen-speciﬁc T lymphocyte responses, an IFNγ-based ELISpot assay was performed as previously described [46], with some modifications. Brieﬂy, an S peptide pool consisting of 15–18-mers (overlapping by 11 amino acids) and spanning the entire S protein of SARS-CoV-2 were synthesized. Spleens of vaccinated BALB/c mice were harvested at 2 weeks post the second dose immunization and plenocytes were isolated. Flat-bottom, 96-well plates were precoated with 10 μg/mL anti-mouse IFNγ Ab (BD Biosciences, USA) overnight at 4°C, and then blocked for 2 h at 37°C. Mouse splenocytes were added to the plate. Then, the peptide pool (2 μg /ml individual peptide) was added to the wells. Phytohemagglutinin (PHA) was added as a positive control. Cells incubated without stimulation were employed as a negative control. After 24 h of incubation, the cells were removed, and the plates were processed in turn with biotinylated IFNγ detection antibody, streptavidin-HRP conjugate, and substrate. When the coloured spots were intense enough to be visually observed, the development was stopped by thoroughly rinsing samples with deionized water. The numbers of the spots were determined using an automatic ELISpot reader and image analysis software (Cellular Technology Ltd.).

### Intracellular cytokine staining (ICS) and ﬂow cytometry

ICS assays were performed as previously described [46], with some modifications. Brieﬂy, mouse splenocytes were added to the plate (2 × 10^6^/well) and then stimulated with the peptide pool (2 μg/mL for individual peptide) for 5 h. PMA and ionomycin (Dakewe Bioengineering, China) were used as a positive control. The cells were incubated with GolgiStop (BD Biosciences, USA) for an additional 6 h at 37°C. Then, the cells were harvested and stained with anti-CD3 (BioLegend), anti-CD4 (BioLegend), and anti-CD8α (BioLegend) surface markers. The cells were subsequently ﬁxed and permeabilized in permeabilizing buffer (BD Biosciences, USA) and stained with anti-mouse anti-IFNγ (BioLegend), anti-TNFα (BioLegend), anti-IL-2 (BioLegend), anti-IL-4 (BioLegend), and anti-IL-10 (BioLegend) antibodies. All ﬂuorescent lymphocytes were gated on a FACSAria ﬂow cytometer (BD Biosciences, USA).

### Animal protection against virus challenge

To evaluate the protection efficacy of vaccine candidates against SARS-CoV-2, a recombinant adenovirus Ad5-hACE2 transducing BALB/c mice model was used. Immunized BALB/c mice were i.n infected with 8 × 10^8^ vp of Ad5-hACE2. Five days later, the transduced mice were challenged with 5 × 10^5^ TCID_50_ of SARS-CoV-2 (hCoV-19/China/CAS-B001/2020, GISAID No. EPI_ISL_514256-7) via the i.n. route. Three days post challenge, mice were euthanized and necropsied. Lung tissues were collected and split into two parts for virus titration and pathological examination. All animal experiments with SARS-CoV-2 challenge were conducted under animal biosafety level 3 (ABSL3) facility in IMCAS.

### qRT-PCR

Mice lung tissues were weighed and homogenized. Virus RNA was isolated from 50-μL supernatants of homogenized tissues using a nucleic acid extraction instrument MagMAX™ Express Magnetic Particle Processor (Applied Biosystems, USA). SARS-CoV-2-speciﬁc quantitative reverse transcription-PCR (qRT-PCR) assays were performed using a FastKing One Step Probe RT-qPCR kit (Tiangen Biotech, China) on a CFX96 Touch real-time PCR detection system (Bio-Rad, USA) according to the manufacturer’s protocol. Two sets of primers and probes were used to detect a region of the N gene of viral genome [49] and a region of E gene of subgenomic RNA (sgRNA) of SARS-CoV-2 [50], respectively, with sequences as follows: N-F, GACCCCAAAATCAGCGAAAT; N-R, TCTGGTTACTGCCAGTTGAATCTG; N-probe, FAM-ACCCCGCATTACGTTTGGTGGACC-TAMRA (where FAM is 6-carboxyﬂuorescein, and TAMRA is 6-carboxytetramethylrhodamine); sgRNA-E-F, CGATCTCTTGTAGATCTGTTCTC; sgRNA-E-R, ATATTGCAGCAGTACGCACACA; sgRNA-E-probe, FAM-ACACTAGCCATCCTTACTGCGCTTCG-TAMRA.

Viral loads were expressed on a log_10_ scale as viral copies/gram after calculation with a standard curve. Viral copy numbers below the limit of detection were set as half the limit of detection.

### Histopathology analysis

Mice lung tissues were fixed in 4% paraformaldehyde, dehydrated, embedded in parafﬁn, and then sectioned. Tissue sections (4 μm) were deparafﬁnized in xylene and stained with haematoxylin and eosin (H&E) for pathological examination, such as peribronchiolitis, interstitial pneumonitis, and alveolitis. Besides, tissue sections were stained with anti-SARS-CoV-2 nucleoprotein antibody (Sino Biological, China) to detect virus infection.

### Statistical analysis

Data are expressed as the means ± standard errors of the means (SEM). For all analyses, *P* values were analysed by unpaired *t*-test. Graphs were generated with GraphPad Prism software.

## Results

### Vaccine design and antigen expression

**Figure 1.**
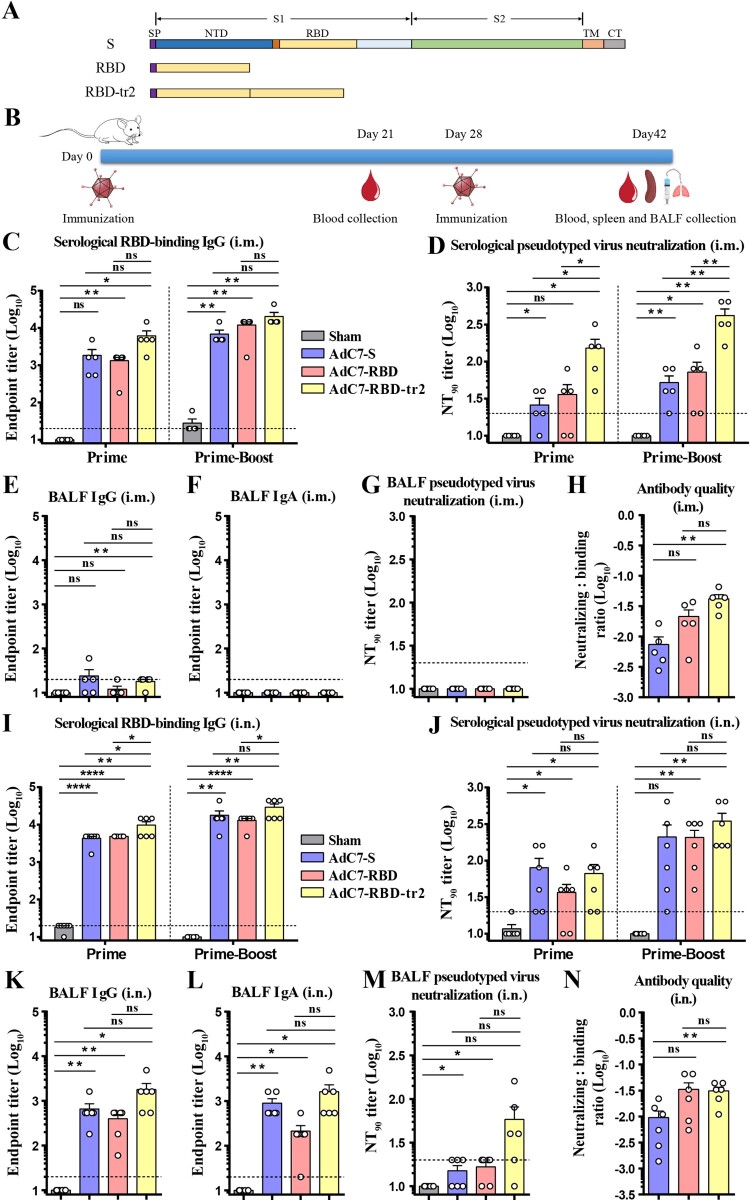
Characterization of the humoral immune responses of BALB/c mice immunized with AdC7 vaccines. (A) Schematic demonstration of antigen constructs of full-length S, RBD, and RBD-tr2. Sequences encoding signal peptide was derived from MERS-CoV S protein. SP, signal peptide; TM, transmembrane anchor; CT, cytoplasmic tail. (B) Schedule of animal experiments. Female BALB/c mice (6–8 weeks old) were immunized with two doses of 1 × 10^11^ vp of AdC7 vaccines through the i.m. or i.n. route, respectively. Sera were collected three weeks post-prime immunization. BALF, sera and spleen were collected two weeks post-boost immunization. (C–H) Antibody responses of BALB/c mice (*n* = 5) vaccinated with AdC7 vaccines via the i.m. route. (C) Measurement of SARS-CoV-2 RBD-binding IgG endpoint titres of serum samples from mice immunized via the i.m. route. Prime indicates serum samples collected at day 21 post the first dose vaccination. Prime-boost indicates serum samples collected at day 14 post the second dose vaccination. (D) Measurement of SARS-CoV-2 pseudotyped virus NT_90_ of serum samples from mice immunized via the i.m. route. (E–G) Measurement of SARS-CoV-2 RBD-binding IgG (E) and IgA (F) endpoint titres and pseudotyped virus NT_90_ (G) of BALF from mice immunized via the i.m. route. (H) Antibody quality of serum samples from two-dose immunized mice via the i.m. route. The ratio is the pseudotyped virus neutralization titre: S protein-binding IgG titre. NT_90_ and S protein-binding titres were shown in Figure 1(D) and Supplementary Figure 3A. (I–N) Antibody responses of BALB/c mice (*n* = 6) vaccinated with AdC7 vaccines via the i.n. route. (I and J) Measurement of SARS-CoV-2 RBD-binding IgG endpoint titres (I) and pseudotyped virus NT_90_ (J) of serum samples from mice immunized via the i.n. route. (K–M) Measurement of SARS-CoV-2 RBD-binding IgG (K) and IgA (L) endpoint titres and pseudotyped virus NT_90_ (M) of BALF from mice immunized via the i.n. route. (N) Antibody quality of serum samples from two-dose immunized mice via the i.n. route. The ratio is the pseudotyped virus neutralization titre: S protein-binding IgG titre. NT_90_ and S protein-binding titres were shown in Figure 1(J) and Supplementary Figure 3B. Data are means ± SEM (standard errors of means). *P* values were analysed with *t*-test (ns, *P *> 0.05; *, *P* < 0.05; **, *P* < 0.01; ****, *P* < 0.0001). The dashed line indicates the limit of detection.

We constructed three replication-incompetent AdC7 vaccines expressing SARS-CoV-2 full-length S, RBD, and RBD-tr2, respectively (Figure 1(A)). The expression cassettes were inserted into the E1 region of the AdC7 vector with E1 and E3 deletion. Western blot was conducted to confirm antigen expression in HEK 293T cells infected with the recombinant adenovirus. All these antigens were detected in the cell lysates (Supplementary Figure 1A). Both monomeric RBD and RBD-tr2 were secreted in the culture supernatants (Supplementary Figure 1B).

### Humoral responses induced by AdC7 vaccines in mice

To evaluate the immunogenicity of the recombinant AdC7 vaccines, groups of female BALB/c mice were immunized on Day 0 and 28 with 1 × 10^11^ vp of AdC7 vaccines via i.m. injection (Figure 1(B)). AdC7 without any transgene (AdC7-empty) was injected as the sham control. Blood samples were collected on Day 21 and 42 (Figure 1(B)). The serological RBD-binding IgG and IgA titres were measured. RBD-specific IgG was robustly induced by AdC7-S, AdC7-RBD, and AdC7-RBD-tr2 (Figure 1(C)), while RBD-specific IgA antibodies were only moderately induced in the mice vaccinated with AdC7-RBD-tr2 (Supplementary Figure 2A). As neutralizing antibodies play a crucial role in protection, pseudotyped virus displaying the SARS-CoV-2 S protein was used to detect the neutralizing antibody titres in the sera of the immunized mice. Two doses of AdC7-S, AdC7-RBD, and AdC7-RBD-tr2 elicited average NT_90_ titres of 52, 72, and 416, respectively (Figure 1(D)). AdC7-RBD-tr2 showed significantly higher neutralization titres compared to the other constructs (Figure 1(D)).

SARS-CoV-2 was transmitted through respiratory droplets and caused lung damage diseases. Specific immune response in the air-way and lung would confer protection against SARS-CoV-2 infection *in situ*. Immunized mice were euthanized for BALF collection. RBD-binding IgG, IgA, and pseudotyped virus NT_90_ were measured for the BALF. As expected, a small amount of RBD-binding IgG was detected in these AdC7 vaccines (Figure 1(E)). Neither RBD-binding IgA nor neutralizing antibodies could be detected in the BALF of mice immunized with AdC7-S, AdC7-RBD, or AdC7-RBD-tr2 (Figure 1(F,G)). In order to compare the quality of antibody, the serological neutralizing:binding ratio was calculated for these three constructs. The results showed AdC7-RBD-tr2 elicited the highest neutralizing:binding ratio, suggesting its advantage of immunofocusing of inducing neutralizing antibodies with potentially reduced ADE risk (Figure 1(H) and Supplementary Figure 3A).

As low levels of RBD-binding and neutralizing antibodies were detected in the BALF of mice immunized with each of the AdC7 vaccines via the i.m. route, we sought to immunize female BALB/c mice intranasally to strengthen mucosal immunity. On Day 0 and 28, the BALB/c mice were vaccinated with 1 × 10^11^ vp of AdC7 via the i.n. route. On Day 21 and 42, serum samples were collected for antibody titration (Figure 1(B)). AdC7-S, AdC7-RBD, and AdC7-RBD-tr2 all elicited substantial RBD-binding IgG and IgA (Figure 1(I) and Supplementary Figure 2B). Two-dose immunizations of AdC7-S, AdC7-RBD, and AdC7-RBD-tr2 induced the pseudotyped virus NT_90_ titres of 210, 207, and 347, respectively (Figure 1(J)). The BALF of mice were also collected on Day 42 for further evaluation. High levels of both RBD-binding IgG and IgA were detected in the BALF of the mice immunized with AdC7-S, AdC7-RBD or AdC7-RBD-tr2 (Figure 1(K,L)). The average endpoint titres of RBD-binding IgG and IgA of AdC7-RBD-tr2 group were 1800 and 1620, respectively, with both values higher than the other two constructs (Figure 1(K,L)). Moreover, pseudotyped virus NT_90_ of the BALF was assessed, with an average of 10, 15, 17, and 58 for sham, AdC7-S, AdC7-RBD, and AdC7-RBD-tr2, respectively (Figure 1(M)). AdC7-RBD-tr2 group showed higher mucosal pseudotyped virus NT_90_ compared to either AdC7-S or AdC7 RBD. In addition, we analysed the antibody quality in mice sera, and demonstrated RBD and RBD-tr2-based vaccines exhibited higher neutralizing: binding ratio than full-length S-based vaccine (Figure 1(N) and Supplementary Figure 3B). In summary, both systemic and mucosal immunity were induced by recombinant vaccines through i.n. vaccination. But for i.m. immunization, mucosal immunity was barely induced, characterized by non-detectable RBD-binding IgA and neutralizing antibodies in BALF. Besides, AdC7-RBD-tr2 showed the advantage of immunofocusing of inducing neutralizing antibodies blocking receptor-binding.

### Cellular responses induced by AdC7 vaccines in mice

**Figure 2.**
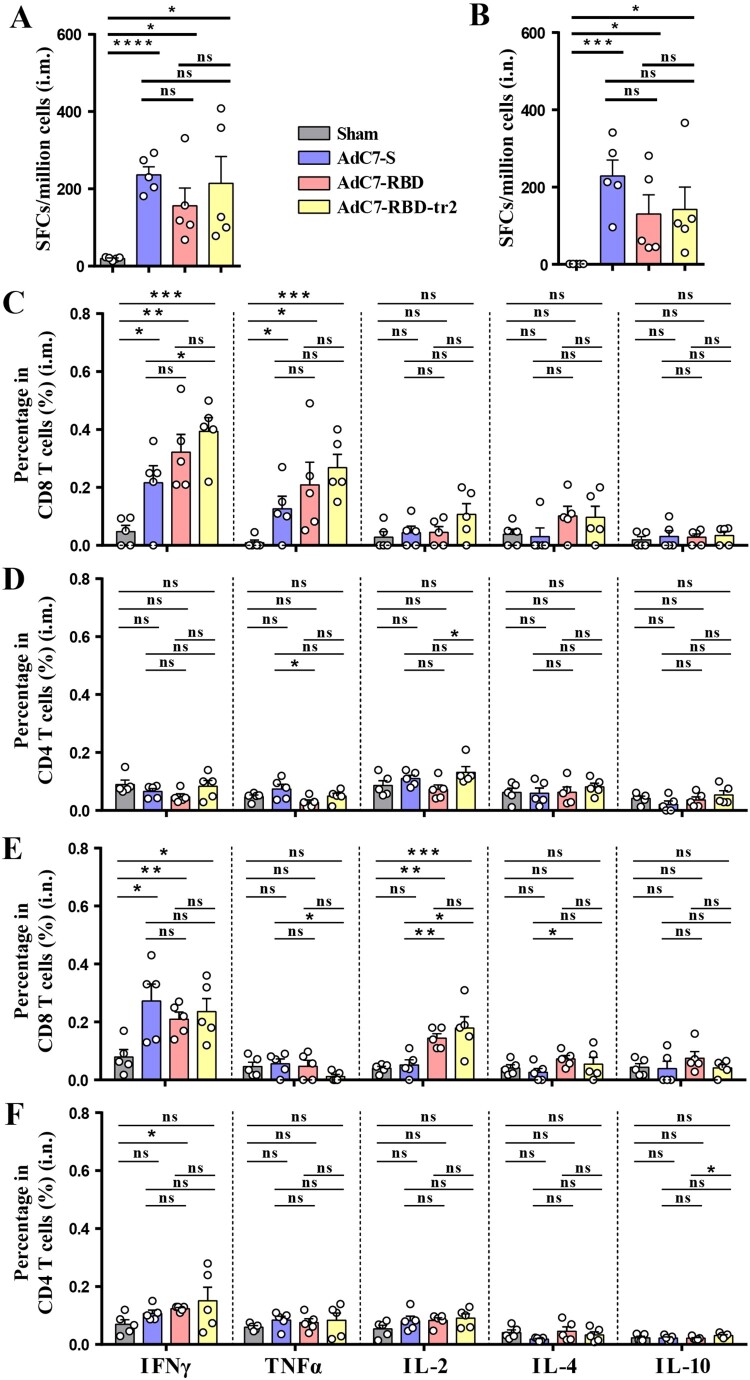
Characterization of the cellular immune responses. BALB/c mice were immunized with 1 × 10^11^ vp AdC7-S, AdC7-RBD, AdC7-RBD-tr2, or Sham (AdC7-empty) as described in Figure 1(B). Mice splenocytes were isolated and analysed by ELISpot and ICS assays. (A and B) ELISpot assays were performed to evaluation of the IFNγ secretion of splenocytes after SARS-CoV-2 S peptides stimulation for mice immunized through the i.m. (A) or i.n. (B) route. (C–F) ICS assays were conducted to analyse the CD8+ and CD4+ T cell responses of mice immunized via the i.m. (C and D) or i.n. (E and F) route. (C and D) Quantiﬁcation of the frequency of IFNγ-, TNFα-, IL-2-, I-4-, and IL-10-producing CD8+ T cells (C) and CD4+ T cells (D) of splenocytes from mice with i.m. immunization. (E and F) Quantiﬁcation of the frequency of IFNγ-, TNFα-, IL-2-, I-4-, and IL-10-producing CD8+ T cells (E) and CD4+ T cells (F) of splenocytes from mice with i.n. immunization. Data are means ± SEM. *P* values were analysed with *t*-test (ns, *P* > 0.05; *, *P* < 0.05; **, *P* < 0.01; ***, *P* < 0.001; ****, *P* < 0.0001).

Aside from humoral immunity, cellular immunity also plays an important role in protection against the SARS-CoV-2 [51,52]. To characterize the cellular immune responses induced by the AdC7 vaccines, the same cohort of immunized BALB/c mice were euthanized on Day 42 (Figure 1(B)). Splenic lymphocytes were harvested and stimulated with an overlapping 11-mer peptide pool spanning the SARS-CoV-2 S protein and analysed by ELISpot and ICS assays. The IFNγ-ELISpot analysis showed the induction of robust T cell responses for mice vaccinated with AdC7-S, AdC7-RBD or AdC7-RBD-tr2 via both i.m. and i.n. routes (Figure 2(A,B)). Additionally, the flow cytometric analyses showed that all these recombinant AdC7 vaccines induced cytotoxic T lymphocytes (CTL), via both i.m. and i.n. routes. The divergent responses associated with IFNγ-, TNFα-, and IL-2-producing cells were observed. Besides, the CD4+ T cell responses cytokines were moderately induced for all these three constructs (Figure 2(D,F)). In contrast, no substantial Th2 cytokines (IL-4 and IL10) production was detected for all these three constructs (Figure 2(D,F)). Another cellular response analyses showed that T-cell responses were induced by 2.5 × 10^10^ vp of AdC7-RBD-tr2 (Supplementary Figure 4). These results demonstrated substantial T-cell responses, especially CTL responses, were induced in mice received AdC7-S, AdC7-RBD, or AdC7-RBD-tr2 vaccines by both i.m. and i.n. routes.

### Protection efficacy of AdC7-RBD-tr2 against SARS-CoV-2 challenge in mice

Since wild-type BALB/c mice are not sensitive to SARS-CoV-2 infection due to the low-binding affinity between mouse ACE2 and S protein, we transduced the Ad5-hACE2 into BALB/c mouse lung via i.n. route to rapidly generate a mouse model [53]. To demonstrate the efficiency of the mouse challenge model, groups of female BALB/c mice were transduced with 8 × 10^8^ vp of Ad5-hACE2 or PBS, followed by challenge with 5 × 10^5^ TCID_50_ SARS-CoV-2 through i.n. route five days later. Mice were necropsied to collect lung tissues for virus titration before SARS-CoV-2 infection (0 days post infection [dpi]) and post-SARS-CoV-2 infection (1, 3, and 5 dpi). The titration results showed that the levels of SARS-CoV-2 genome RNA (gRNA) gradually decreased in the PBS-transduced mice (Supplementary Figure 5A). Whereas high levels of virus gRNA sustained from 1 to 5 dpi in Ad5-hCAE2-transduced mice (Supplementary Figure 5A). In addition, virus sgRNA could not be detected in PBS-transduced mice, while high levels of sgRNA were observed in Ad5-hCAE2-transduced mice (Supplementary Figure 5B). These results indicated SARS-CoV-2 entered host cells and efficiently replicated. This mouse challenge model was used in the following evaluation of protection efficacy of AdC7-RBD-tr2.

**Figure 3.**
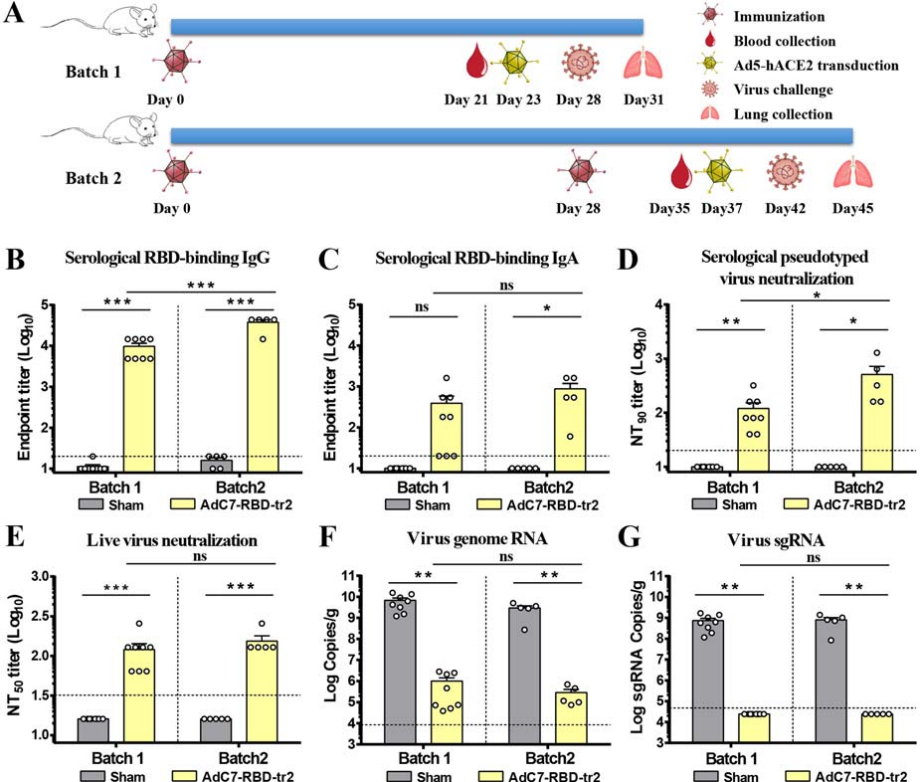
Protective efficacy of AdC7-RBD-tr2 against SARS-CoV-2. (A) Immunization and challenge schedule. Female BALB/c mice (6–8 weeks old) of batch 1 (*n* = 8) and batch 2 (*n* = 5) received one dose or two-dose of 1 × 10^11^ vp of AdC7-RBD-tr2 through the i.n. route, respectively. The same dose of sham vaccine (AdC7-empty) group was i.n. infected as the control. Prior to SARS-CoV-2 challenge, blood samples from vaccinated mice were collected for antibodies titration. At five days post-Ad5-hACE2 transduction, mice were i.n. challenged with 5 × 10^5^ TCID_50_ SARS-CoV-2. Animals were euthanized and necropsied on 3 dpi. and lung tissues were harvested for virus titration and pathological examination. (B and C) Measurement of SARS-CoV-2 RBD-binding IgG (B) and IgA (C) endpoint titres of sera. (D) Measurement of pseudotyped virus NT_90_ of sera. (E) Measurement of real SARS-CoV-2 neutralizing antibody titres (NT_50_) of sera. (F and G) SARS-CoV-2 titration from lung tissues by qRT-PCR probing virus gRNA (F) and sgRNA (G). Data are means ± SEM. *P* values were analysed with *t*-test (ns, *P* > 0.05; *, *P* < 0.05; **, *P* < 0.01; ***, *P* < 0.001). The dashed line indicates the limit of detection.

Given the fact that AdC7-RBD-tr2 elicited the most robust RBD-binding IgG, IgA, and neutralizing antibodies, AdC7-RBD-tr2 vaccine was chosen for further protection efficacy against SARS-CoV-2 challenge. Two batches of BALB/c mice were immunized with one-dose (batch1) or two-dose (batch2) of 1 × 10^11^ vp of AdC7-RBD-tr2 via i.n. route (Figure 3(A)). AdC7-RBD-tr2 inoculation induced BALB/c mice producing substantial titres of RBD-binding IgG, IgA (Figure 3(B,C)) and pseudotyped virus-neutralizing antibody (Figure 3(D)). Additionally, live virus neutralization assay revealed a mean titre of 120 (one-dose) and 154 (two dose), respectively, upon AdC7-RBD-tr2 vaccination (Figure 3(E)). These mice were challenged with 5 × 10^5^ TCID_50_ SARS-CoV-2 five days post transduction of Ad5-hACE2. Three days post challenge lungs of mice were harvested for virus titration and pathologic analysis. High levels of viral gRNA were observed with a mean titre of 9.83 log_10_ RNA copies/g in mice lung in the one dose (batch1) sham group (Figure 3(F)). Whereas, the low level of viral gRNA was observed in the AdC7-RBD-tr2-vaccinated mice with a mean titre of 6.00 log_10_ RNA copies/g (Figure 3(F)). In the prime-boost groups of mice (batch2), the mean titre of viral gRNA (log_10_) in mice lung per gram was 9.47 and 5.46 for sham and AdC7-RBD-tr2, respectively (Figure 3(F)). Because a fraction of viral RNA in the lung was probably originated from input challenge virus, levels of sgRNA were also measured to quantify the live virus. The sgRNA were generated in the infected cells during virus replication but were absent in the virions [54]. As a result, high levels of sgRNA were observed with a mean titre of 8.87 and 8.91 log_10_ RNA copies/g in the sham group with the one-dose and two-dose regimens, respectively. Encouragingly, viral sgRNA could not be detected in any lung samples from both one and two doses of AdC7-RBD-tr2-vaccinated mice (Figure 3(G)), indicating the complete protection efficacy of AdC7-RBD-tr2 vaccine.

**Figure 4.**
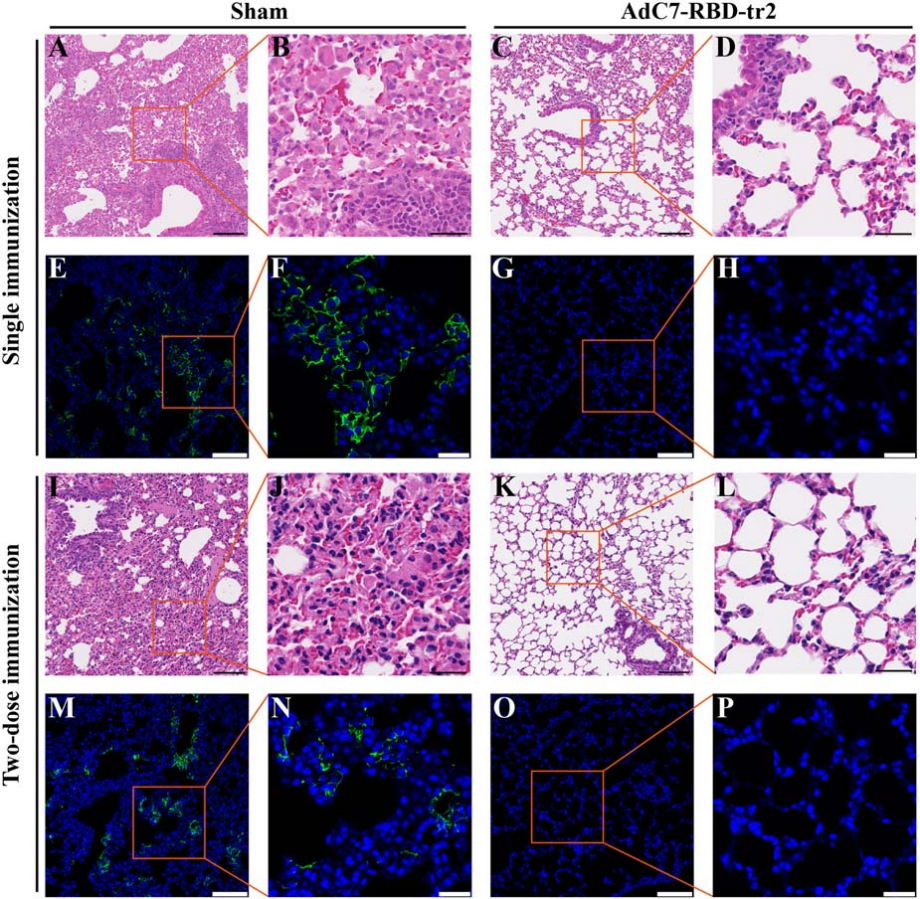
Protection against lung infection and lesions by AdC7-RBD-tr2. Mice lung tissues were fixed in 4% paraformaldehyde, embedded in parafﬁn, and then sectioned. Tissue sections (4 μm) were stained with H&E or anti-SARS-CoV-2 nucleoprotein antibody for pathological examination and virus probing. (A–H) Histopathology and immunofluorescence analysis of lung tissue sections from batch 1 mice with single immunization. (I–P) Histopathology and immunofluorescence analysis of lung tissue sections from batch 2 mice with two doses immunization. (A–D and I–L) Images of lung pathology from sham group (A, B, I, and J) and AdC7-RBD-tr2 group (C, D, K, and L). Both low magniﬁcations (A, C, I, and K) and high magniﬁcations (B, D, J, and L) are shown. (E–H and M–P) Images of immunofluorescence from sham group (E, F, M, and N) and AdC7-RBD-tr2 group (G, H, O, and P). Both low magniﬁcations (E, G, M, and O) and high magniﬁcations (F, H, N, and P) are shown. Scale bar in low magniﬁcations images, 100 μm. Scale bar in high magniﬁcations images, 30 μm.

In order to further evaluate vaccine protection against lung damage by SARS-CoV-2, histopathological analysis was performed. The results showed mice from sham group developed apparent viral pneumonia characterized by thickened alveolar walls, vascular congestion, and inﬂammatory cell inﬁltration (Figure 4(A,B,I,J)), whereas a marked attenuation of pathological damage and inﬂammatory response were seen in the lung tissues of mice vaccinated with AdC7-RBD-tr2 (Figure 4(C,D,K,L)). More importantly, immunofluorescence analysis of lung section stained with anti-SARS-CoV-2 NP antibody revealed the virus presence in the lung of the sham group (Figure 4(E,F,M,N)), but not in that of AdC7-RBD-tr2 group (Figure 4(G,H,O,P)). In summary, these results demonstrated the AdC7-RBD-tr2 vaccine robustly elicited mice immune responses against SARS-CoV-2 and confer nearly sterilizing immunity against SARS-CoV-2 infection.

### Immune responses and protection efficacy conferred by low dose of AdC7-RBD-tr2

**Figure 5.**
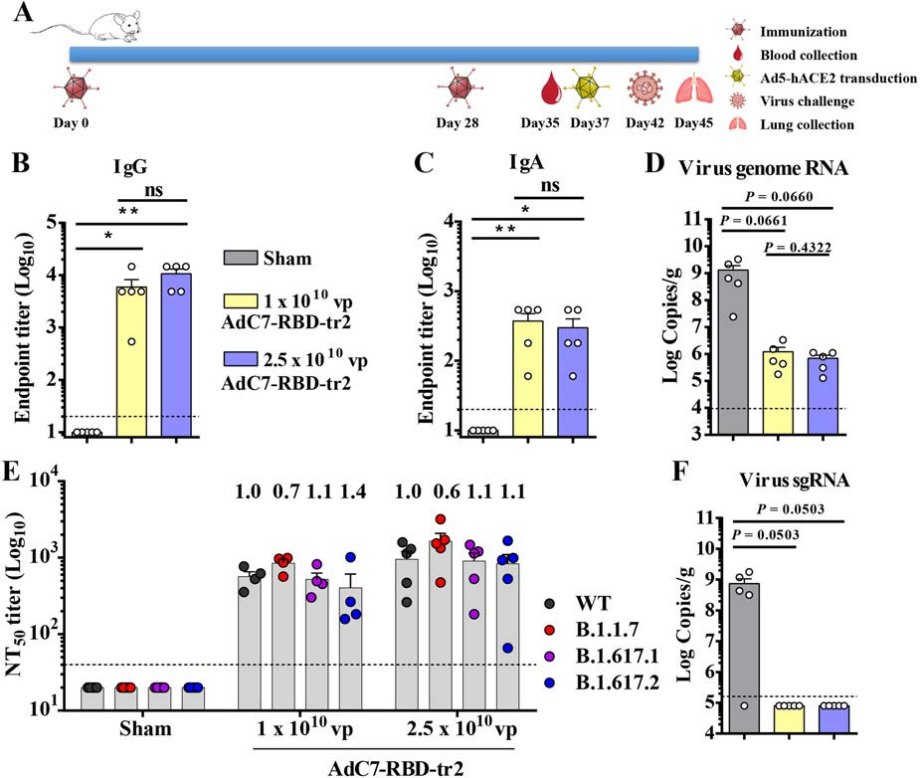
Protective efficacy of low dose of AdC7-RBD-tr2 against SARS-CoV-2. (A) Immunization and challenge schedule. Female BALB/c mice (6–8 weeks old, *n* = 5) received two-dose of 1 × 10^10^ or 2.5 × 10^10^ vp of AdC7-RBD-tr2 through the i.n. route. Prior to SARS-CoV-2 challenge, blood samples from vaccinated mice were collected for antibodies titration. At five days post-Ad5-hACE2 transduction, mice were i.n. challenged with 5 × 10^5^ TCID_50_ SARS-CoV-2. Animals were euthanized and necropsied on 3 dpi. and lung tissues were harvested for virus titration. (B and C) Measurement of SARS-CoV-2 RBD-binding IgG (B) and IgA (C) endpoint titres of sera. (D and F) SARS-CoV-2 titration from lung tissues by qRT-PCR probing virus gRNA (D) and sgRNA (F). (E) Measurement of neutralizing antibody activity against pseudotyped virus displaying early Wuhan-1 reference strain: NT_50_ against variant. Data are means ± SEM. *P* values were analysed with *t*-test (ns, *P* > 0.05; *, *P* < 0.05; **, *P* < 0.01). The dashed line indicates the limit of detection.

To evaluate the immunogenicity of low dose of AdC7-RBD-tr2, groups of female BALB/c mice were immunized with two-dose of 1 × 10^10^ vp or 2.5 × 10^10^ vp of AdC7-RBD-tr2 via the i.n. route (Figure 5(A)). Serological IgG subclass and IgA RBD-binding antibodies were titrated. The results showed that RBD-binding IgG1, IgG2a, IgG, and IgA were all substantially induced (Figure 5(B,C) and Supplementary Figure 6).

Recently, the emergence of SARS-CoV-2 variants was of highly concerned. Several (VOCs) showed increased transmissibility and virulence. Some of the VOCs were reported to reduce vaccine efficacy [55,56]. To analyse the neutralization of AdC7-RBD-tr2-elicited sera against SARS-CoV-2 variants, a panel of pseudotyped viruses displaying variant S protein of B.1.1.7 (Alpha), B.1.617.1 (Kappa), or B.1.617.2 (Delta) lineage were used to measure the 50% pseudovirus neutralization titre. Pseudotyped virus displaying S protein of Wuhan-1 reference strain was used as the control. The results showed that B.1.1.7-spike pseudovirus did not reduce, but slightly enhanced the sensitivity to AdC7-RBD-tr2-elicited sera (Figure 5(E)). In contrast, the serological neutralizing activity against B.1.617.1 and B.1.617.2 slightly decreased with a fold ranging from 1.1 to 1.4 (*p* > 0.05) as compared with neutralization of Wuhan-1 reference strain (Figure 5(E)).

To evaluate the protection efficacy provided by low dose of AdC7-RBD-tr2, the above-immunized BALB/c mice were transduced with Ad5-hACE2. Two doses of AdC7-RBD-tr2 do not elicit sera that neutralize Ad5 before Ad5-hACE2 transduction (Supplementary Figure 7). The transduced mice were challenged with SARS-CoV-2. Lungs of mice were collected at 3 dpi for virus titration. The mean titres of viral gRNA (log_10_) RNA and sgRNA for sham groups were 9.11 and 8.87 copies/g (Figure 5(D,F)). For mice immunized with 1 × 10^10^ vp or 2.5 × 10^10^ vp of AdC7-RBD-tr2 via i.n. route, the mean titres of gRNA(log_10_) were 6.08 and 5.84 copies/g (Figure 5(D)), with the reduction of virus titre more than 3 log. The SARS-CoV-2 sgRNA was completely undetectable in both 1 × 10^10^ and 2.5 × 10^10^ vp groups (Figure 5(F)). These results demonstrated that low dose (1 × 10^10^ vp and 2.5 × 10^10^ vp) of AdC7-RBD-tr2 is protective in mice against SARS-CoV-2 infection.

## Discussion

To control COVID-19 pandemic, a broad vaccine pipeline worldwide is necessary. Vaccine candidates were developed with different platforms, vectors, antigens, doses, vaccination strategies, vaccination routes, and mechanisms. ChAdOx1 nCoV-19 [28], Gam-COVID-Vac [14], Ad5-nCoV [29], Ad26.COV2.S [17], and ChAd-SARS-CoV-2-S [16] all express full-length S protein of SARS-CoV-2. Previously, our team had developed a protein subunit COVID-19 vaccine (ZF2001) based on the RBD-tr2 designation, which induced significantly higher humoral responses than monomeric RBD protein vaccine in animals [11]. The clinical results showed ZF2001 was well tolerated and immunogenic. In March 2021, ZF2001 had been approved for emergency use in China and Uzbekistan. Here, we constructed a recombinant AdC7 vaccine expressing RBD-tr2 and demonstrated that it induced robust humoral responses with high neutralizing: binding ratio, an indication of immune focusing of the antibody responses. In addition, sera from AdC7-RBD-tr2-vaccinated mice preserved neutralizing activity against the circulating strains, including the B.1.1.7, B.1.617.1, and B.1.617.2.

Upon immunization of AdC7-RBD-tr2 in mice via the i.m. or i.n. route, similar levels of RBD-binding and neutralizing antibodies titres were observed. However, mucosal immunity was barely induced through i.m. immunization of AdC7-RBD-tr2, but could be effectively activated through i.n. immunization, characterized by RBD-binding IgA and neutralizing antibodies detected in BALF. Cellular immunity also plays an important role in protection against the SARS-CoV-2 [51,52]. We observed substantial T-cell responses in mice induced by AdC7-RBD-tr2 through both i.m. and i.n. route, in particular, the CTL responses. AdC7-RBD-tr2 conferred mice complete protection against SARS-CoV-2 without infection enhancement or immunopathological exacerbation. Vaccination via i.n route would be advantageous in easy administration and better protection in the respiratory tract, an ultimate goal to abrogate SARS-CoV-2 transmission.

Both AdC7-RBD-tr2 and ZF2001 are based on the RBD-tr2 as the antigen. ZF2001 is a protein subunit vaccine containing RBD-tr2 protein adjuvanted with aluminium hydroxide. Both ZF2001 and AdC7-RBD-tr2 induced similar level of neutralizing antibodies in mice [57]. However, compared with the protein subunit vaccine [11], AdC7-based vaccine induced a more pronounced T cell responses in mice in the present study.

ChAdOx1 nCoV-19 is a chimpanzee adenovirus-vectored vaccine. One dose of 6 × 10^9^ vp ChAdOx1 nCoV-19 induced humoral responses and Th1-like T-cell responses in mice through i.m. vaccination [27]. A single i.m. immunization of 10^10^ vp Ad26.COV2.S induced neutralizing antibody production and cellular immunity that was polarized towards Th1 IFN-γ in mice [58]. A single i.m. or i.n. immunization of 10^7^, 10^8^, or 10^9^ vp of Ad5-nCoV induced S-binding antibody and neutralizing antibody in mice. Cellular responses were induced by one-dose i.m. or i.n. immunization of 10^8^ vp Ad5-nCoV [29]. It should be noted that comparing the neutralizing antibody levels for various vaccines is difficult due to different methods and lack of standardized benchmark. AdC7-RBD-tr2 is based on chimpanzee type 7 vector with the advantage of low level of pre-existing immunity. A single dose of 1 × 10^11^ vp AdC7-RBD-tr2 immunization through i.n. route induced neutralization antibody production and conferred mice complete protection against SARS-CoV-2. Two-dose of 1 × 10^11^ vp, 2.5 × 10^10^ vp and 1 × 10^10^ vp of AdC7-RBD-tr2 were immunogenic in mice and conferred protection against SARS-CoV-2.

There were several limitations in this study. Firstly, the immunogenicity of AdC7-RBD-tr2 with a single dose of 1 × 10^10^ vp or lower was not evaluated, which will be conducted for further development. Secondly, we evaluated the protection efficacy of AdC7-RBD-tr2 with a Ad5-hACE2-transduced mouse model. Additional severe models, such as hACE-2 knockin mice, should be used to validate the protective effects of AdC7-RBD-tr2 in the pre-clinical stage. Thirdly, the potential ADE induced by vaccine candidates was not detected by experiment in this study. During the stage of revision, two teams reported that several anti-N-terminal domain (NTD) antibodies targeting the specific epitopes of NTD mediated *in vitro* infection enhancement. Besides, some anti-RBD neutralizing antibodies also showed FcγR-mediated enhancement of virus infection *in vitro*, but not *in vivo* [34,35] As AdC7-RBD-tr2 express dimeric RBD protein, without induction of anti-NTD antibodies, which suggests its advantage of reducing the potential ADE risk. Fourthly, we found that humoral responses were not strongly boosted by a second dose immunization. Both single dose and two-dose of AdC7-RBD-tr2 conferred mice complete protection against SARS-CoV-2. The antibody production kinetics of single dose and two-dose immunization should be studied in the further development. Finally, it was reported that vaccination could potently suppress virus burden and ameliorate histopathological damages in the lower respiratory tract but usually not the upper tract [59]. In the present study, we demonstrated the protection of AdC7-RBD-tr2 vaccine in lung. Further studies should investigate the vaccine protection in the upper tract via i.n. route.

Collectively, AdC7-RBD-tr2 is a potential new vaccine candidate with single-dose injection, cheap, and easy to produce (according to the chimpanzee adenovirus COVID-19 vaccine ChAdOx1 nCoV-19 by AstraZeneca). AdC7-RBD-tr2 induced neutralizing antibody responses, CTL responses and conferred protection in respiratory tract. Our results support AdC7-RBD-tr2 as vaccine candidate to move forward for pre-clinical and clinical trials for prophylaxis of COVID-19.

## Supplementary Material

clean_copy_of_supplementary_material.docx

## Data Availability

All data associated with this study are available from the corresponding author upon reasonable request.
