# Supplementary material for: Recombinant chimpanzee adenovirus AdC7 expressing dimeric tandem-repeat spike protein RBD protects mice against COVID-19
Source: Emerg Microbes Infect. 2021 Aug 12;10(1):1574–88. doi: 10.1080/22221751.2021.1959270 (PMC8366625; doi:10.1080/22221751.2021.1959270)
Supplement: clean_copy_of_supplementary_material.docx [file TEMI_A_1959270_SM9942.docx]

**Supplemental material**

Recombinant chimpanzee adenovirus AdC7 expressing dimeric tandem-repeat spike protein RBD protects mice against COVID-19

Kun Xu^a,b#^, Yaling An^c#^, Qunlong Li^d#^, Weijin Huang^e#^, Yuxuan Han^c^, Tianyi Zheng^b^, Fang Fang^d^, Hui Liu^d^, Chuanyu Liu^f^, Ping Gao^b^, Senyu Xu^b^, Xueyuan Liu^g^, Rong Zhang^f^, Xin Zhao^h,j^, William J. Liu^i^, Yuhai Bi^h,j^, Youchun Wang^e^, Dongming Zhou^k^, Qinghan Wang^d^**^*^**, Wenli Hou^d^**^*^**, Qianfeng Xia^a^**^*^**, George F. Gao^b,h,i^**^*^**, Lianpan Dai^a,h^**^*^**

*Corresponding author. Email: dailp@im.ac.cn (L.D.); gaof@im.ac.cn (G.F.G.); xiaqianfeng@hainmc.edu.cn (Q.X.); hwl@kangh.com (W.H.); wqh@kangh.com (Q.W.)

Supplementary Figure 1


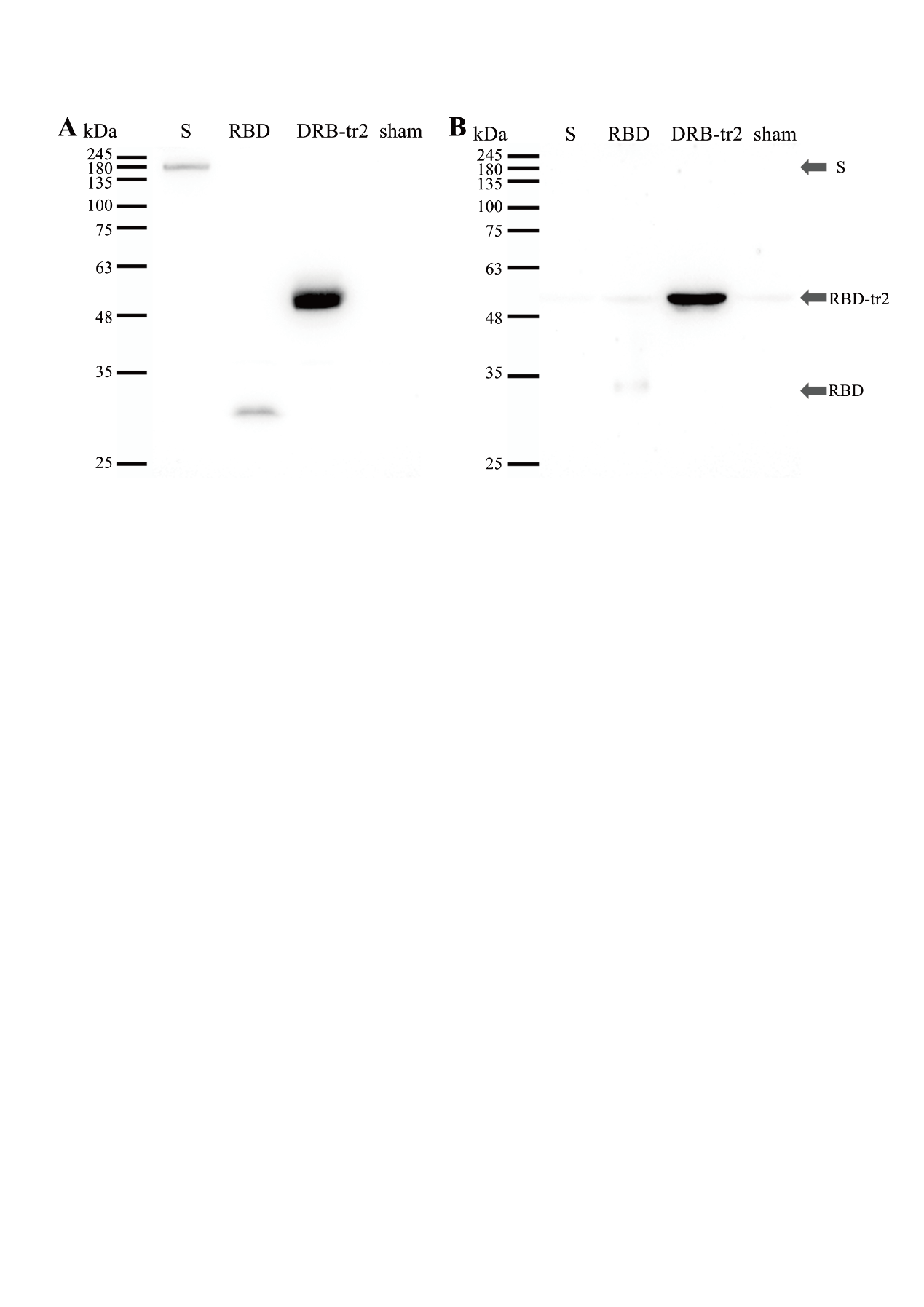


Supplementary Figure 1. Analysis of transgene expression by western blot. HEK293T cells were infected with AdC7-S, AdC7-RBD, AdC7-RBD-tr2 or AdC7-empty (sham). Antigen proteins were probed in cell lysates (A) and supernatants (B).

Supplementary Figure 2


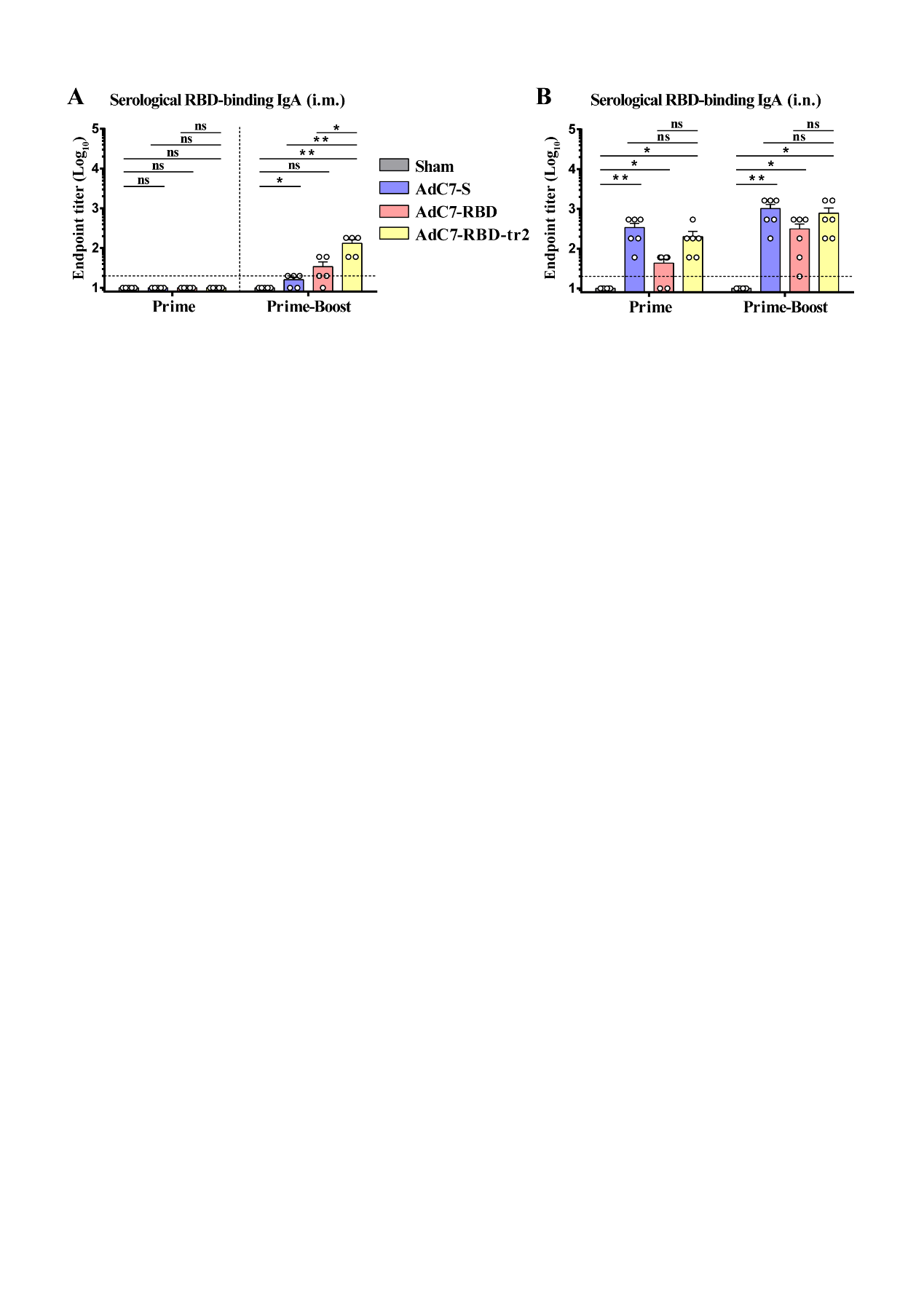


Supplementary Figure 2. Induction of RBD-binding IgA in BALB/c mice immunized with AdC7 vaccines. Measurement of SARS-CoV-2 RBD-binding IgA endpoint titers of serum samples from mice immunized via the i.m. (A) and i.n. (B) route. Prime indicates serum samples collected at day 21 post the first dose vaccination. Prime-Boost indicates serum samples collected at day 14 post the second dose vaccination. Data are means ± SEM. *P* values were analyzed with *t* test (ns, *P* > 0.05; *, *P* < 0.05; **, *P* < 0.01). The dashed line indicates the limit of detection.

Supplementary Figure 3


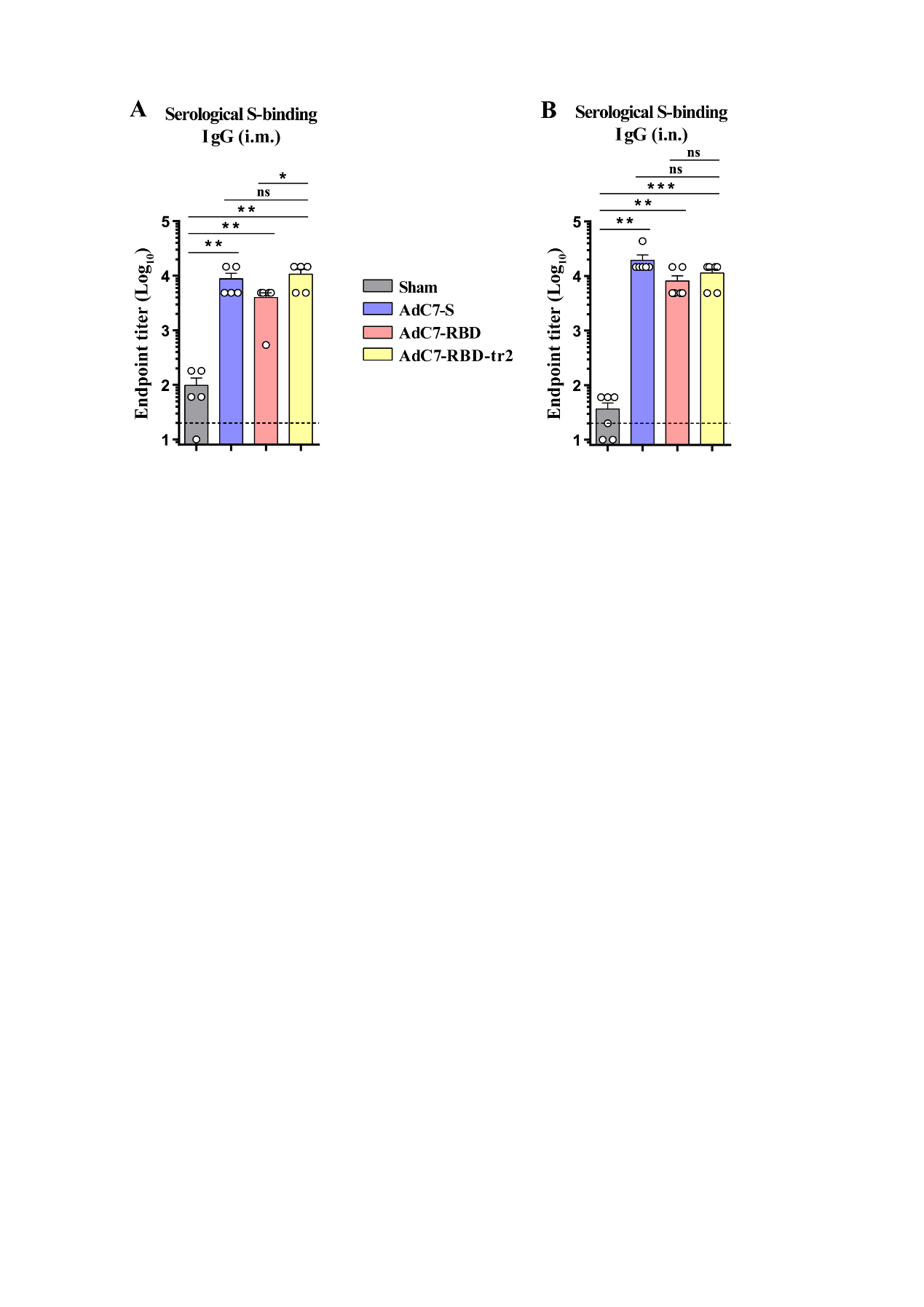


Supplementary Figure 3. Induction of SARS-CoV-2 S protein-binding IgG in BALB/c mice immunized with AdC7 vaccines. Measurement of SARS-CoV-2 full-length S protein-binding IgG endpoint titers of serum samples from mice immunized two doses of AdC7 vaccines via the i.m. (A) and i.n. (B) route. Data are means ± SEM. *P* values were analyzed with *t* test (ns, *P* > 0.05; *, *P* < 0.05; **, *P* < 0.01; ***, *P* < 0.001). The dashed line indicates the limit of detection.

Supplementary Figure 4


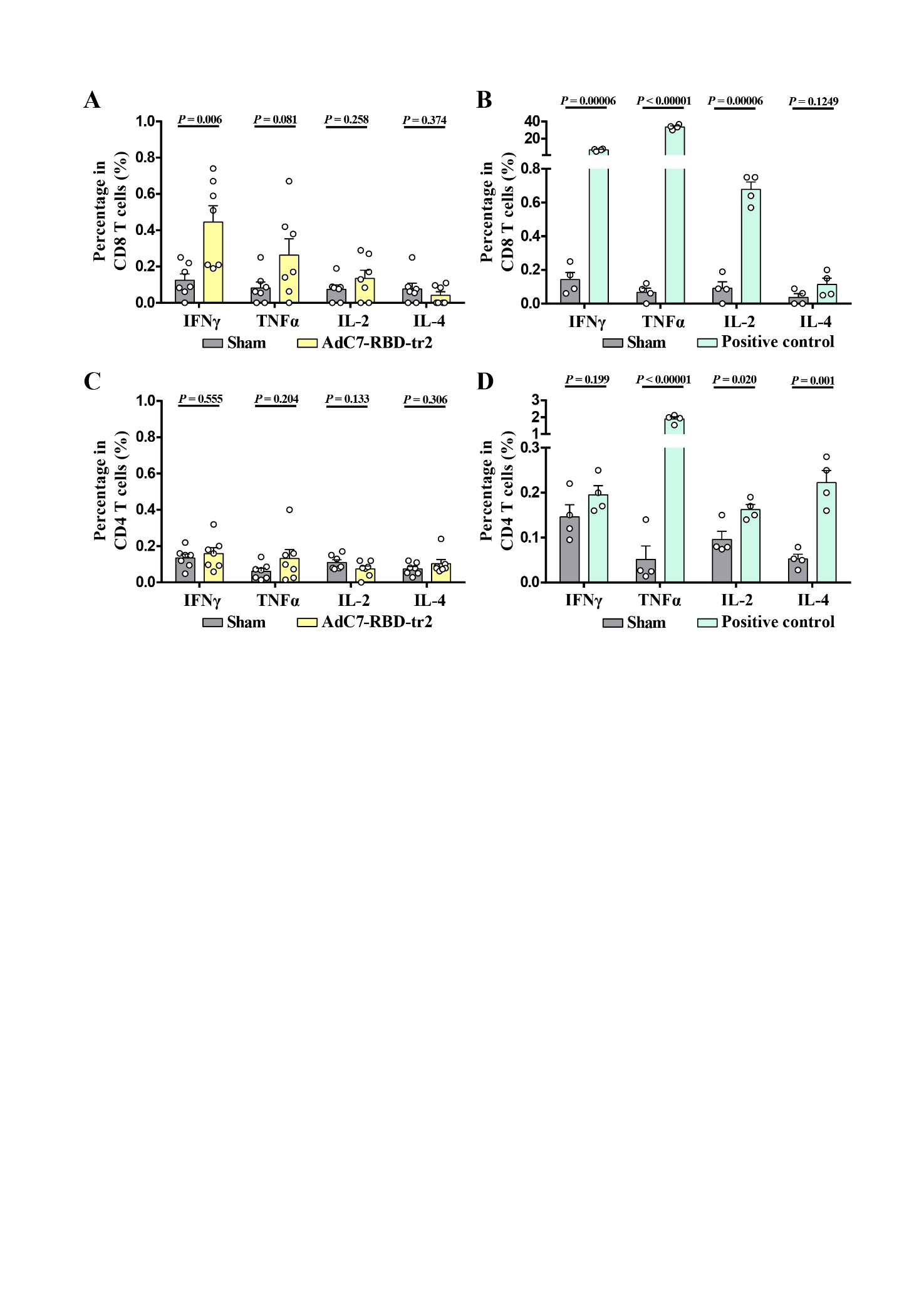


Supplementary Figure 4. Characterization of the cellular immune responses induced by AdC7-RBD-tr2. Female BALB/c mice (n = 7) were i.m. immunized with 2.5 x 10^10^ vp AdC7-RBD-tr2 on Day 0 and 28. Animals were euthanized and necropsied on Day 42 and spleen tissues were harvested. Splenocytes were isolated and analyzed by ICS assays. (A and C) Quantiﬁcation of the frequency of IFNγ-, TNFα-, IL-2- and I-4-producing CD8+ T cells (A) and CD4+ T cells (C) of splenocytes. All the splenocytes samples were elicited by SARS-CoV-2 RBD peptide pool. (B and D) Quantiﬁcation of the frequency of IFNγ-, TNFα-, IL-2- and I-4-producing CD8+ T cells (B) and CD4+ T cells (D) of splenocytes. Splenocytes samples (n=4) from sham group of mice were analyzed with stimulation by positive control (PMA+Ionomycin) and SARS-CoV-2 RBD peptide pool, respectively. Data are means ± SEM. *P* values were analyzed with *t* test.

Supplementary Figure 5


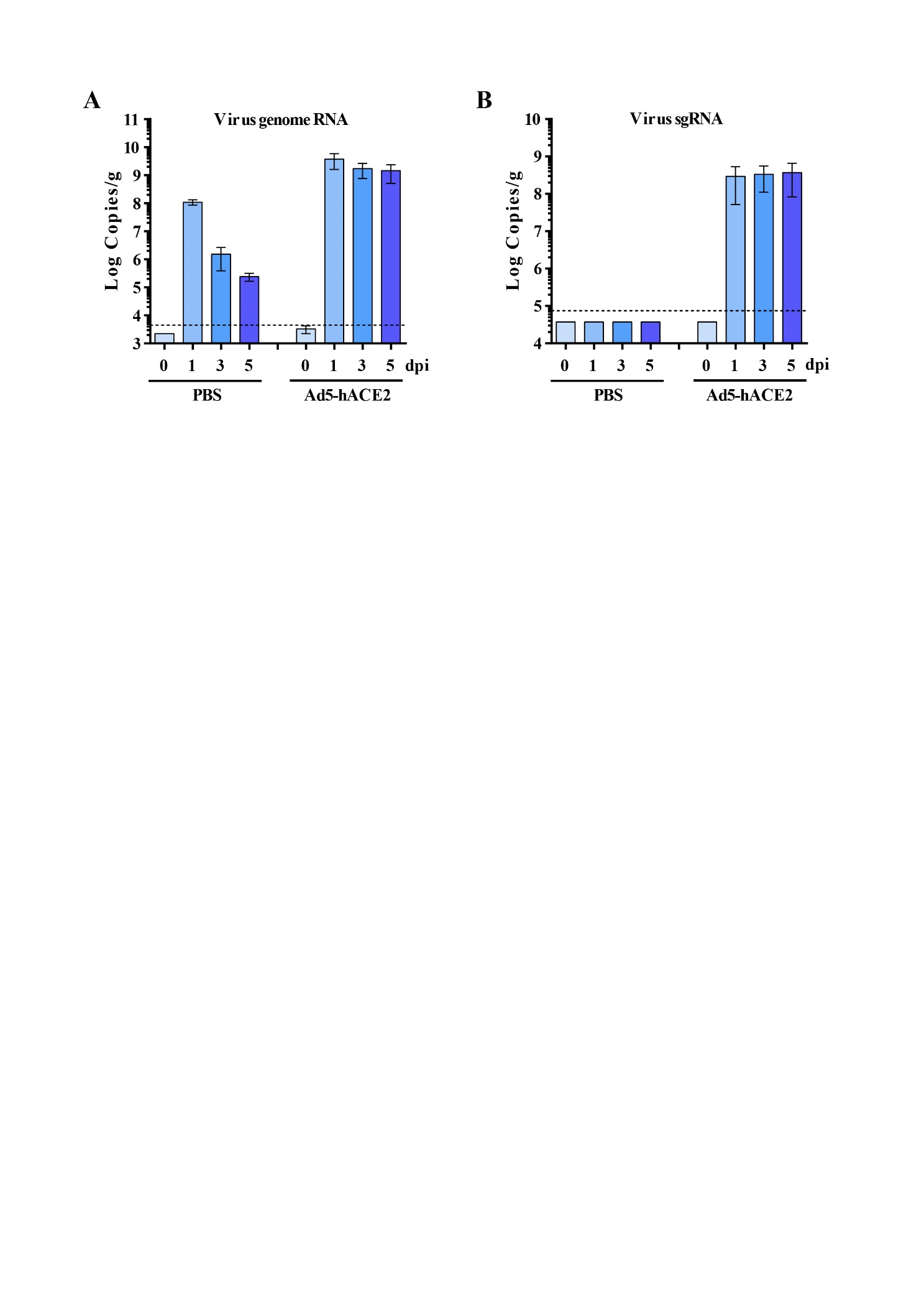


Supplementary Figure 5. Validation of mouse challenge model. Groups of female BALB/c mice (n = 12) were i.n. transduced with 8 x 10^8^ vp of Ad5-hACE2 or PBS, followed by challenged with 5 x 10^5^ TCID_50_ SARS-CoV-2 through i.n. route five days later. Mice were necropsied to collect lung tissues for virus titration before SARS-CoV-2 infection (Day 0) and post SARS-CoV-2 infection (Day 1, 3 and 5). (A and B) SARS-CoV-2 titration from lung tissues by qRT-PCR probing virus gRNA (A) and sgRNA (B). Data are means ± SEM.

Supplementary Figure 6


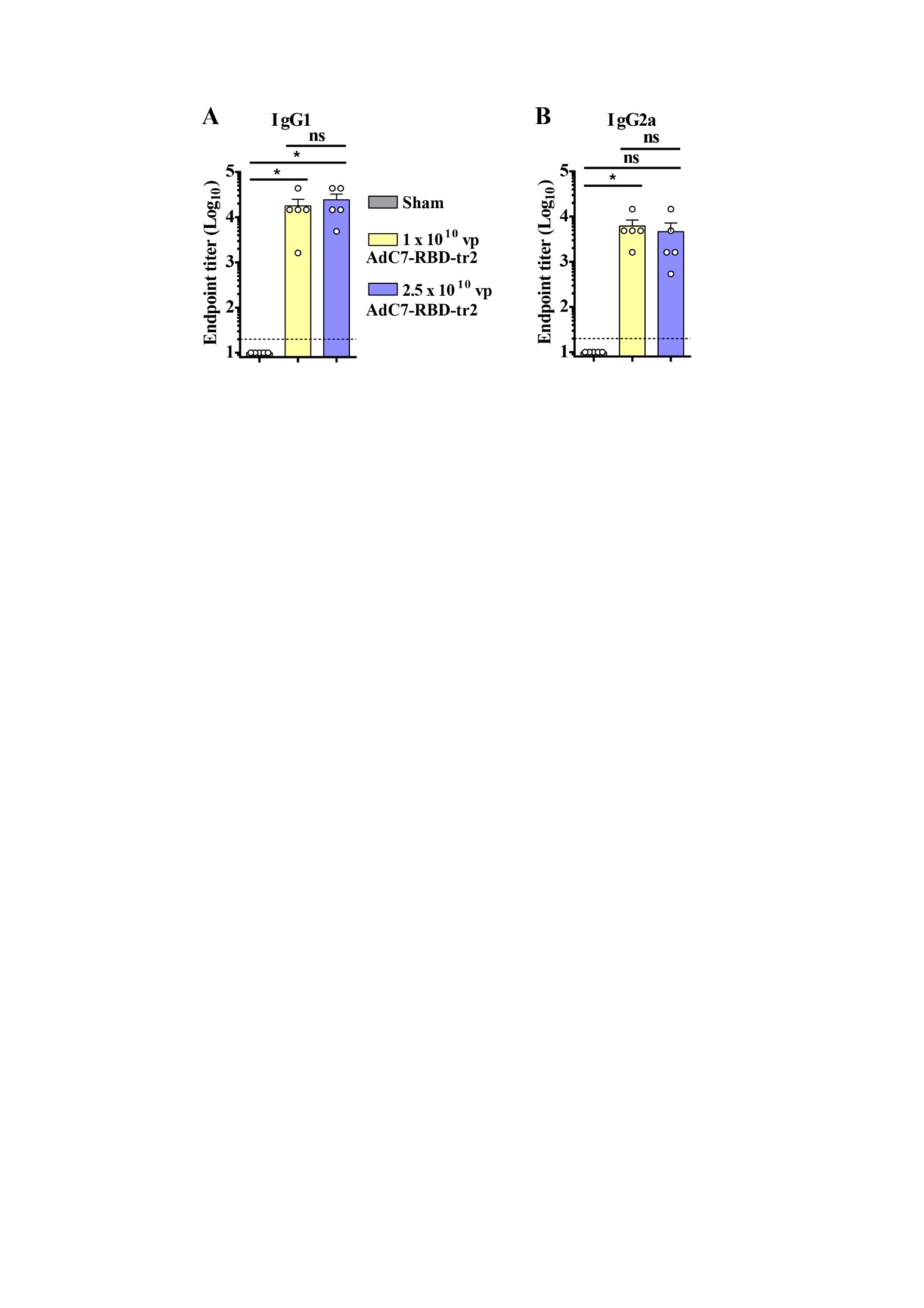


Supplementary Figure 6. SARS-CoV-2 RBD-binding IgG1 and IgG2a titers. Measurement of SARS-CoV-2 RBD-binding IgG1 (A) and IgG2a (B) endpoint titers of mice sera. Data are means ± SEM. *P* values were analyzed with *t* test (ns, *P* > 0.05; *, *P* < 0.05). The dashed line indicates the limit of detection.

Supplementary Figure 7


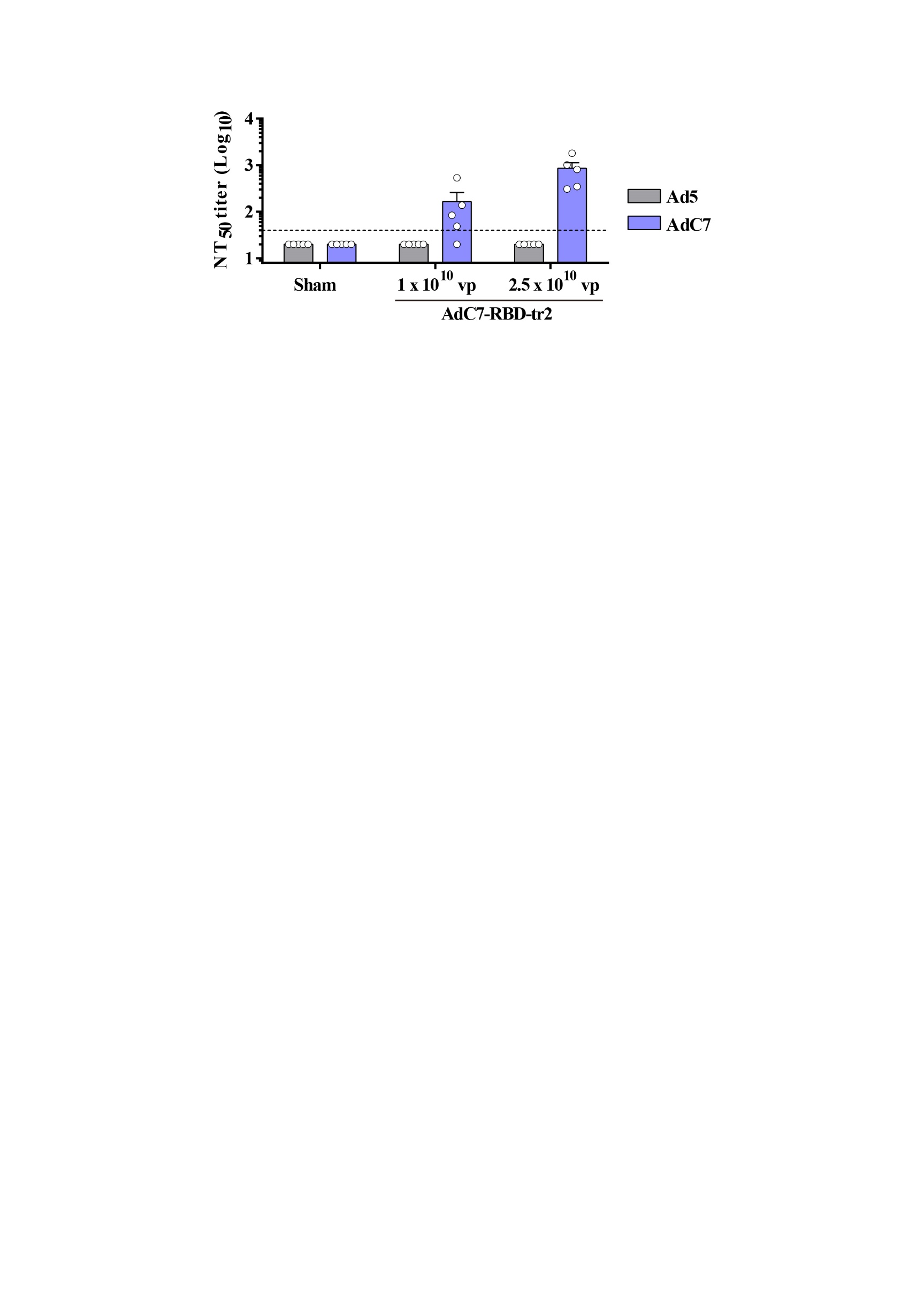


Supplementary Figure 7. Evaluation of the neutralizing activity against AdC7 and Ad5. Recombinant Ad5 expressing GFP and recombinant AdC7 expressing GFP were used in neutralizing activity titration. The sera sample were from AdC7-RBD-tr2-immunized BALB/c mice as depicted in Figure 5A. Data are means ± SEM. The dashed line indicates the limit of detection.
